# Gut Microbiota Alteration in Healthy Preterm Infants: An Observational Study from Tertiary Care Center in India

**DOI:** 10.3390/microorganisms13030577

**Published:** 2025-03-03

**Authors:** Prabavathi Devarajalu, Jogender Kumar, Sourabh Dutta, Savita Verma Attri, Jayakanthan Kabeerdoss

**Affiliations:** 1 Pediatric Biochemistry Unit, Department of Pediatrics, Post Graduate Institute of Medical Education & Research (PGIMER), Chandigarh 160012, India; prabavathidevaraj@gmail.com (P.D.); attrisavi@yahoo.co.in (S.V.A.); 2 Newborn Unit, Department of Pediatrics, Post Graduate Institute of Medical Education & Research (PGIMER), Chandigarh 160012, India; jogendrayadv@gmail.com (J.K.); sourabhdutta1@gmail.com (S.D.); 3 Pediatric Biochemistry Unit, Department of Pediatric Medicine, Advance Pediatric Center (APC), Post Graduate Institute of Medical Education & Research (PGIMER), Sector 12, Chandigarh 160012, India

**Keywords:** preterm infants, gut, microbiota, NICU, 16S rRNA gene sequencing, probiotics, sex, mode of delivery, gestational age, postnatal age, calprotectin, India

## Abstract

Various prenatal and postnatal factors such as gestational age, mode of delivery, sex, antibiotic exposure, feeding type, duration of feed and other exposures associated with the hospital environment can drive the formation of gut microbiota. In the current study, we examined the role of all these factors in the gut microbiota of healthy Indian preterm infants admitted to NICU in the first four weeks of life. Preterm neonates admitted to the NICU from April 2023 and October 2023 were recruited and fecal samples were collected weekly once beginning from the seventh day till the 30th day of life. 16s rRNA gene sequencing was performed on the NovaSeq 6000 platform. The PICRUSt2 tool was used to predict the functional profiles of the gut microbiome. A total of 61 samples were collected from 16 preterm infants. Alpha and beta diversity showed the administration of probiotics, postnatal age, mode of delivery, and sex of infants as major contributors to altered microbial diversity in preterm infants. The MaAsLin2 analysis showed that the supplementation of probiotics increased *Bifidobacterium* levels. PICRUSt2 analysis revealed that probiotic supplementation increased the bacterial genes responsible for bile acid metabolism and glycosphingolipid synthesis. Probiotics and postnatal age are responsible for alterations of the gut microbial composition in healthy preterm infants.

## 1. Introduction

The gut microbiome progressively evolves throughout infancy. However, preterm infants often require prolonged hospitalization and undergo various treatment regimens, including antibiotics, medications, and altered feeding practices. All these factors promote an imbalance in the microbial composition in the intestine and alter bacterial diversity [1]. The immaturity of the intestine of preterm infants and improper colonization of bacteria lead to dysbiosis. This favors the colonization of pathogenic facultative anaerobes, placing preterm infants at heightened risk of developing necrotizing enterocolitis (NEC) and other long-term morbidities [2,3].

Early life gut microbiota is modulated by various prenatal and postnatal factors such as gestational age, mode of delivery, sex, antibiotic exposure, type of feeds, feeding methods, duration of feeds, and other exposures associated with the neonatal intensive care unit (NICU) environment [4,5].

The transmission of blood-borne pathogens to the gut lumen is frequently observed in premature infants due to their underdeveloped intestinal barrier [6]. Nosocomial pathobionts are prevalent in the fecal matter of these infants and can be identified prior to their entry into the bloodstream. Additionally, blood-borne pathogens are associated with alterations in the gut microbial composition of hospitalized preterm infants [7]. Probiotics have been demonstrated to lower the risk of blood-borne infections in premature infants [8]. The administration of probiotics to preterm neonates has nearly become standard practice in neonatology. However, the impact of probiotics on the modulation of intestinal bacterial composition, in conjunction with other prenatal factors influencing premature infants, remains unclear.

Nearly 50% of the global preterm births belong to low middle-income countries (LMIC) [9]. Studying gut bacteria in preterm with different geographic locations including LMIC will aid in designing future microbiome-targeted therapies and reduce preterm-related morbidities. The ecology succession of the gut microbiome in preterm infants from underdeveloped countries is relatively less studied. In addition, the contribution of prenatal and postnatal factors on gut microbiota composition has not been detailly studied in LMIC including India. In this context, we examined the role of all these factors and fecal calprotectin in the gut microbiota of healthy Indian preterm infants admitted to NICU in the first four weeks of life.

## 2. Materials and Methods

### 2.1. Samples and Data Collection

The current study is an observation study. The recruitment of preterm infants admitted to NICU from April 2023 and October 2023 was based on the following inclusion criteria: (1) gestational age between 26 and 32 weeks; (2) birth weight less than 1500 g and (3) postnatal age not exceeding 72 h. A baby’s life expectancy of less than a week, congenital malformations, refusal to provide samples, and lack of parental consent were among the exclusion criteria. Demographic information of the participants was gathered prospectively up until discharge in predifined proforma. The probiotics group consisted of samples were collected from infants after probiotic supplementation. The non-probiotics group comprised of fecal samples were collected prior to the administration of probiotics.

Fecal samples were collected in sterile containers every week, beginning on the seventh day and continuing until the thirtieth day of life. Informed consents were obtained from mothers before the enrollment of neonates. The stool samples were immediately stored at −20 °C in the NICU itself and later were transferred to the laboratory within the next 24 h where they were stored in an ultra-deep freezer until further processing. The study was approved by the Institutional Ethics Committee (IEC) and was conducted in accordance with the Declaration of Helsinki.

### 2.2. Calprotectin Assay

Fecal calprotectin was estimated using a commercially available quantitative enzyme-linked immunoassay method (Epitope Diagnostics Inc., San Diego, CA, USA).

### 2.3. 16S rRNA Gene Sequencing

Genomic DNA was isolated using approximately 100 milligrams of fecal specimens utilizing DNeasy PowerSoil Pro Kits in accordance with the protocols delineated by the manufacturer (Qiagen GmbH., Hilden, Germany). The purity of the extracted DNA was evaluated via 1% agarose gel electrophoresis. The concentration of DNA was determined utilizing the Qubit DNA HS assay (Invitrogen, Waltham, MA, USA). Each sample designated for sequencing was prepared following Illumina 16S rRNA gene sequencing library protocols targeting the V3-V4 region. PCR amplicons were examined on 2% agarose gel electrophoresis for detection. AMPure XP beads (Beckman Coulter, Brea, CA, USA) were used to clean up amplified PCR products. Libraries were generated using the Nextera XT library preparation kit (Illumina San Diego, CA, USA). The concentration and integrity of the libraries were assessed for fragment distribution utilizing the capillary electrophoresis apparatus (5300 Fragment Analyzer System, Agilent, Santa Clara, CA, USA). DNA libraries were subsequently sequenced in the NovaSeq 6000 platform (Illumina, San Diego, CA, USA) to conduct 16s rRNA gene sequencing employing a 2 × 300 bp paired-end configuration.

Primer sequences were removed from demultiplexed fastq files using the cutadapt tool. All the downstream sequencing analyses were performed in the R statistical program. The fastq files were imported into the DADA2 v1.10.1 [10] to generate a unique amplicon sequence variant (ASV) table. Taxonomy was assigned to each ASV against a SILVA database, version 138, at 99% sequence similarity. The generated ASVs were filtered and the taxa counts present in more than 20% of the samples were retained; the rest of the taxa were removed from further analysis.

### 2.4. Microbiome Composition and Statistical Analysis

Alpha diversity metrics, including the observed ASV and Shannon index, were calculated using the Phyloseq package v1.48.0 [11]. A one-way analysis of variance (ANOVA) was employed to evaluate the disparities in diversity indices among samples obtained across four weeks. The Wilcoxon rank-sum test was utilized to assess the differences in diversity indices among preterm infants, those receiving probiotics, and other demographic groups. Beta diversity metrics were computed employing the vegan package v2.6-10 within the R programming environment. A permutational analysis of variance (PERMANOVA) was conducted using the adonis2 function to ascertain statistical significance by fitting the distance data alongside metadata through 999 permutations.

Differential bacterial abundance between the variables was evaluated by the Linear discriminant analysis (LDA) effect size (LEFSe) analysis with LDA 3 setting in the microbiomeMarker package v1.10.0 [12,13]. Microbiome Multivariable Association with Linear Models (MaAsLin2) [14] is used to identify gut microbiota associated with clinical variables. Subjects were used as a random effect; the rest of the variables were treated as fixed effects and the analysis was performed using default setting.

PICRUSt2 (Phylogenetic Investigation of Communities by Reconstruction of Unobserved States) analysis was carried out to predict metabolic pathways from the filtered ASVs and was annotated using the KEGG database [15]. Statistical analyses were performed in the R software using various packages including vegan [16], microbiomeseq [17], ggplot2 [18], and ggpicrust2 [19]. A linear regression model using the LinDA package v0.2.0 was used to identify significant metabolic pathways [20]. Multiple testing correction was performed in both differential abundance testing and MaAsLin2, with q < 0.05 considered significant.

## 3. Results

The study participant characteristics are mentioned in Table 1. A total of 61 samples were collected from 16 preterm infants. Before giving birth, all the mothers received prenatal steroids, but only one mother received antibiotics. All the infants received donor or expressed breast milk. Only one infant suffered an episode of feeding intolerance and a sample collected during that time was removed from analysis. None of the neonates have sepsis, NEC, or any other serious complications.

### 3.1. Alpha- and Beta-Diversity of Preterm Infants

**Figure 1:**
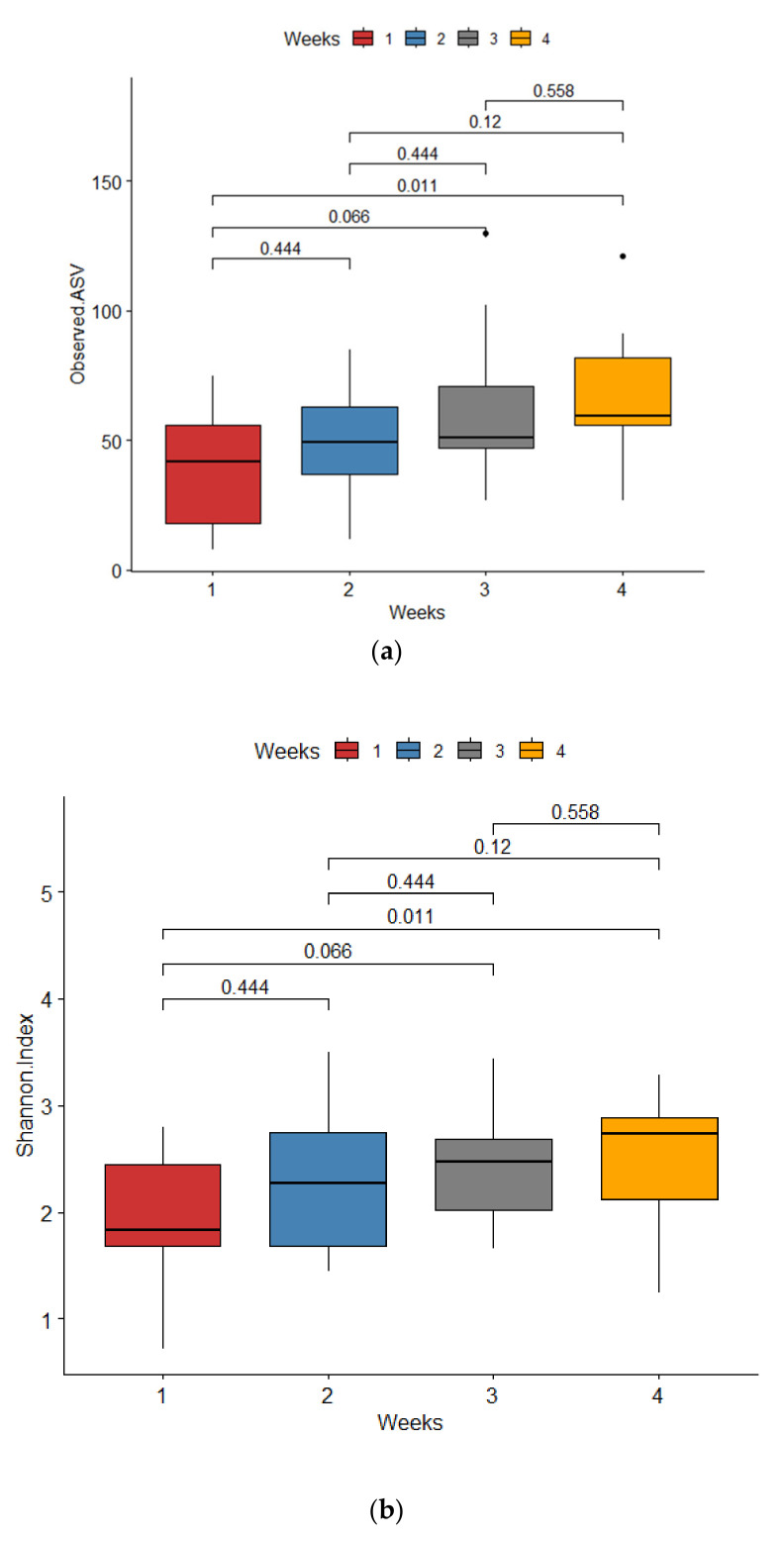
Alpha diversity metrics: (**a**) observed ASVs and (**b**) Shannon index in first four weeks of preterm infants.

**Figure 2:**
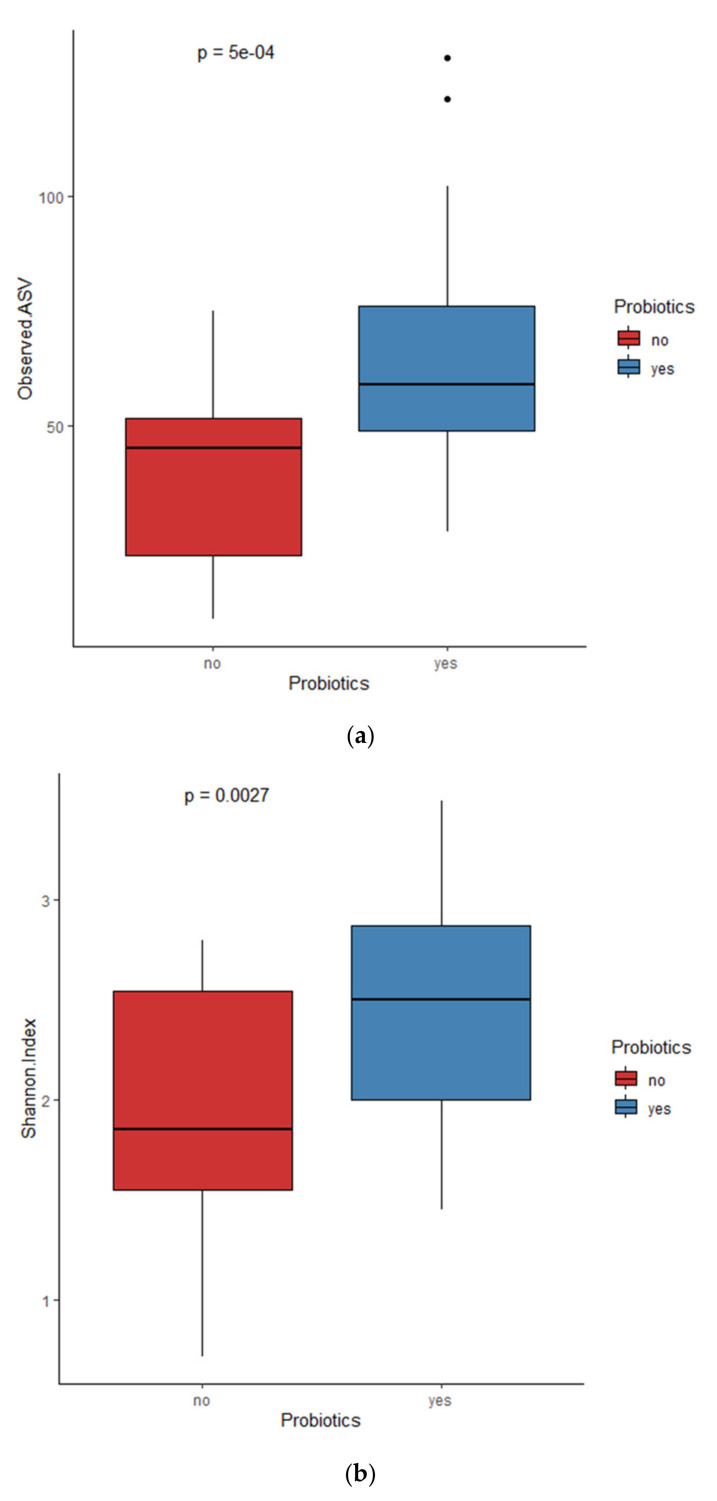
(**a**) Observed ASVs and (**b**) Shannon index on samples before and after probiotics supplementation.

**Figure 3:**
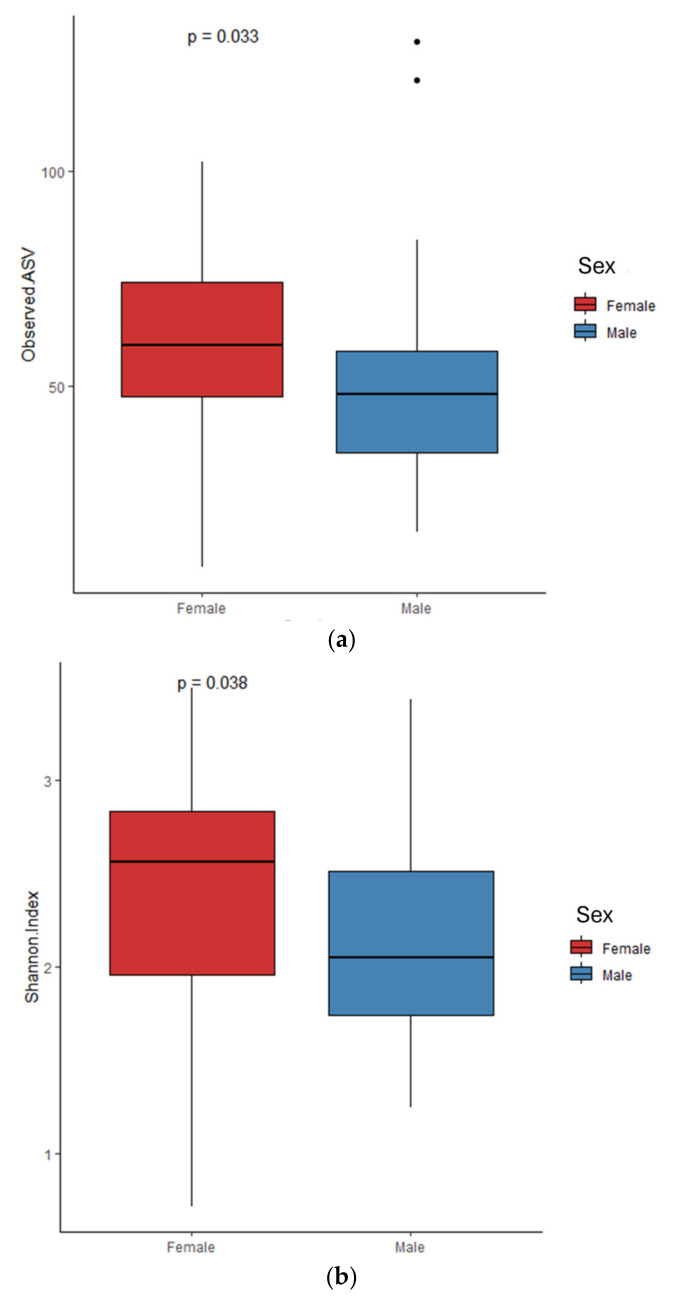
(**a**) Observed ASVs and (**b**) Shannon index on samples based on sex.

The observed ASV and Shannon indices used to evaluate microbial richness and evenness showed probiotics supplementation, mode of delivery, sex, and the timing of sample collection during the first four weeks were associated with alpha diversity metrics. The observed ASV and Shannon index gradually increased in the samples collected from the first to the fourth weeks (Figure 1). The alpha diversity indices were significantly higher in the samples from infants after the initiation of probiotic supplementation compared to those collected prior to supplementation (Figure 2) as well as between female and male preterm neonates (Figure 3). Even though the Shannon index was reached significantly between the samples from the preterm infants delivered via C-section and those delivered vaginally, the observed ASV showed only a trend (*p* = 0.057) [Supplementary Figure S1]. The observed ASV and Shannon values did not differ between the infants born at <28 weeks and ≥28 weeks of pregnancy as well as between the infants born at <1000 g and ≥1000 g of birthweight (Supplementary Figures S2 and S3).

**Figure 4:**
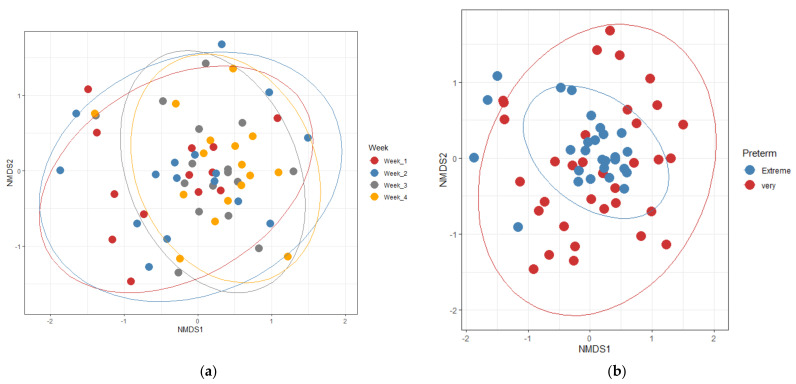

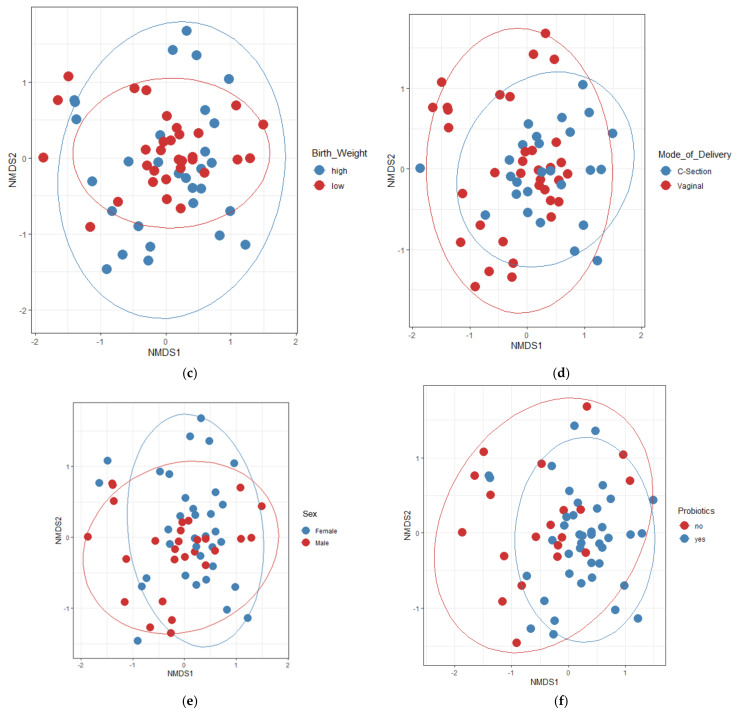
Nonmetric multidimensional scaling (NMDS) plot generated based on Bray–Curtis distance. Each sample represents a dot. (**a**) Colored according to weeks. (**b**) Preterm. (**c**) Birth weight. (**d**) Mode of delivery. (**e**) Sex. (**f**) Probiotics.

The PERMANOVA multivariate analysis calculated using Bray–Curtis and Jaccard methods showed that microbial composition was influenced by postnatal weekly samples, gestation age, probiotic supplementation, mode of delivery, birth weight, and sex (Table 2 and Supplementary Table S1). The weighted UniFrac method showed significance for groups comparing probiotic supplementation, model of delivery, gestational age and birth weight but not for sex and postnatal age. (Supplementary Table S2). Nonmetric multidimensional scaling (NMDS) plotted using Bray–Curtis distances discriminates third- and fourth-week samples from the first- and second-week samples (Figure 4).

### 3.2. Taxa Associated with Clinical Variables

**Figure 5:**
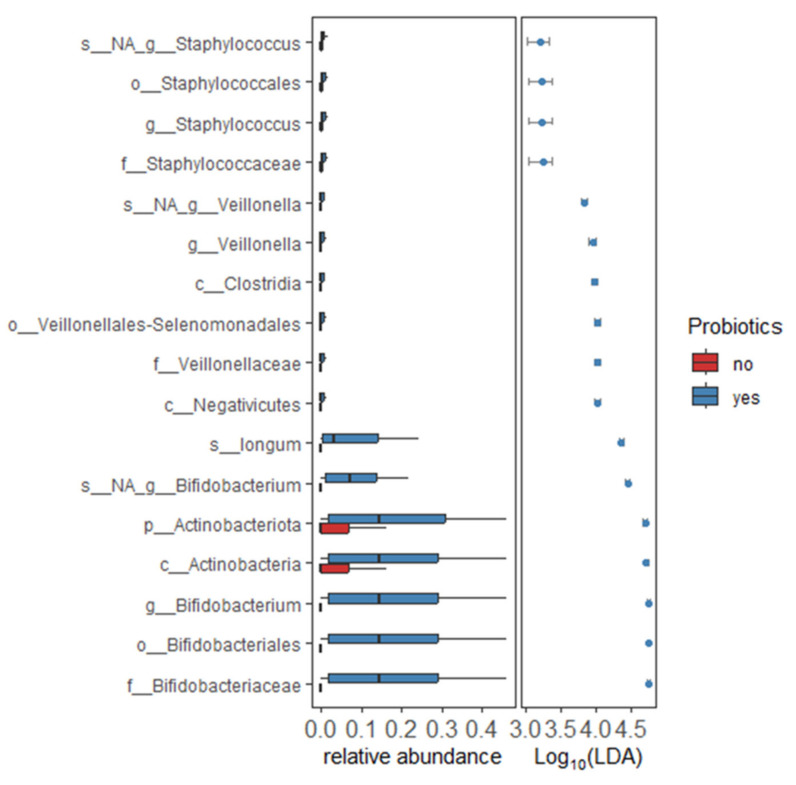
LEfSe analysis identified differential microbial abundance between samples collected before and after probiotic supplementation.

The LEfSe analysis revealed an increased abundance of the *Bifidobacterium* genus including *Bifidobacterium longum*, *Veillonella*, and *Staphylococcus* genus in the samples collected before and after probiotic supplementation (Figure 5). Phylum *Bacteroidota* is significantly enriched in very preterm infants and depleted in extremely preterm infants (Supplementary Figure S4). Similarly, the abundance of unclassified *Bacteroides* species and *Bacteroides* genus were higher in preterm infants with weights above 1000 g compared to low birth weight (Supplementary Figure S5) as well as between the preterm infants delivered by vaginal and cesarean section (Supplementary Figure S6). Unclassified *veillonella* was increased in the samples collected from the first week to the fourth-week samples (Supplementary Figure S7).

A linear model multivariate analysis, the MaAsLin2 analysis confirmed the LEfSe findings that increased the abundance of *Bifidobacterium* genus including *Bifindobacterium longum* in the samples collected after probiotic supplementation compared to the non-probiotic samples (Table 3 and Supplementary Figure S8). *Klebsiella*, *Clostridium sensu stricto 1*, *Enterobacteriaceae*, *Enterococcus*, *Staphylococcus*, *Bifidobacterium*, *Escherichia-Shigella*, and *Veillonella* were increased from the samples collected from different time points from first week to fourth week (Table 4). A weak association was found between calprotectin and *Staphylococcus epidermidis* as well as unclassified *Enterococcus* when fitting postnatal age as a covariate in the linear regression analysis. However, no association was found for calprotectin alone in the MaAsLin 2 analysis. No significant bacteria taxa were identified between the preterm infants with birth weight of <1000 g and ≥1000 g, between the extremely and very preterm infants, between cesarean section and vaginal delivery, and between the female and male preterm infants.

### 3.3. Functional Metabolic Pathways

**Figure 6:**
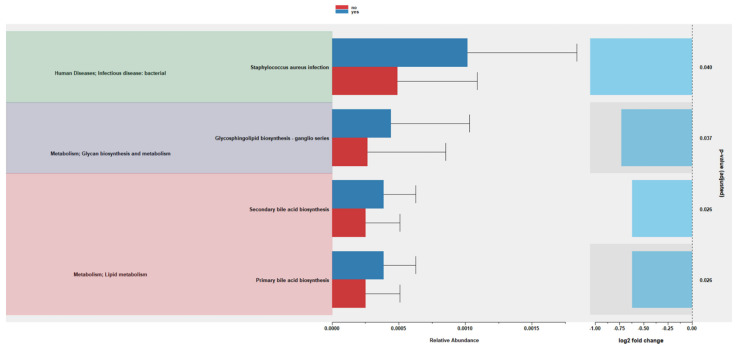
Bar plot depicting significant metabolic pathways identified by functional prediction analysis in samples of probiotic supplementation compared to non-supplemented samples. Grey color represents confidence interval.

Picurst2 predicted metabolic pathways and a differential abundance testing using LinDA analysis showed an increase in bacterial genes for primary bile acid and secondary bile acid metabolism as well as glycosphingolipid synthesis in samples collected after probiotic supplementation compared to the non-supplemented samples (Figure 6). None of the parameters, including gestational age, birth weight, mode of delivery, and sex, were associated with significant functional metabolic changes. Differential abundance using the LefSE analysis identified that the pathways involved in the bacterial invasion of epithelial cells and endocytosis were significantly different between the probiotic supplementation and non-supplemented samples (Supplementary Table S3). No significant pathways were identified by the MaAsLin2 analysis.

## 4. Discussion

The gut microbiota is dynamic and driven by multiple interaction factors in the NICU environment. The present study found that the microbial alterations in the gut of preterm infants during the first month of life were significantly influenced by postnatal age and probiotic supplementation. None of the other covariates including gestation age, mode of delivery, birth weight, and sex were associated with bacterial counts.

The alpha diversity indices gradually increased from the first week to the fourth week. This showed that the progression of postnatal age leads to a gradual increase in bacterial ASV/OTUs and richness in the first month of life [21,22]. The MaAsLin 2 analysis revealed that *Klebsiella*, *Clostridium sensu stricto 1*, *Enterobacteriaceae*, *Enterococcus*, *Staphylococcus*, *Bifidobacterium*, *Escherichia-Shigella*, and *Veillonella* were associated with postnatal age. These dominant taxa were previously reported in the fecal samples of preterm infants [4,23].

The observed ASV and Shannon’s index showed the highest significance when comparing the non-probiotic and probiotic supplemented samples. In contrast, the significance threshold was relatively lower for comparisons between the postnatal samples and between the sex groups. The significance threshold is much higher for probiotics compared to other clinical covariates examined for alpha and beta diversity analysis. The differential abundance analysis showed an increase in *Bifidobacterium breve*, *Veillonella*, and *Staphylococcus* in the samples collected after probiotics supplementation. The MaAsLin 2 analysis confirmed the abundance of these bacterial taxa increased with probiotic supplementation. Furthermore, the probiotic treatment results increased the abundance of *Klebsiella*, *Clostridium sensu stricto 1*, *Enterobacteriaceae*, and *Escherichia-Shigella* and decreased the abundance of *Acinetobacter* in the gut of the preterm infants. Previous probiotic intervention studies in infants born prematurely showed that there was an increase in these bacterial taxa following the supplementation period [24,25]. Probiotic supplementation leading to a reduction in potentially pathogenic *Acinetobacter* bacteria was consistent across the studies [26,27].

The beta diversity dissimilarity analysis using Bray–Curtis and Jaccard showed that probiotic supplementation, mode of delivery, and sex were significantly associated with microbial changes in premature babies. Further differential abundance analysis by LEfSe identified that *Lactobacillus*, *Streptococcaceae*, and *Bifidobacterium breve* were increased in the infants born by cesarean section, whereas *Bacteroides* was increased in the gut of the infants born by vaginal delivery. No bacterial taxa were identified in the linear regression analysis for the mode of delivery. This suggests that the bacterial taxa identified by the LEfSe analysis between mode of delivery could be influenced by probiotic supplementation, as shown in PERMANOVA. The mode of delivery caused changes in the composition of the gut microbiota that were more noticeable right after birth. This mode of delivery-related microbial alterations diminishes over time and might not sustain on advancing age of infants. [28]. Similarly, further differential abundance analysis by LEfSe between sexes identified *Lactobacillaceae*. However, no significant bacterial taxa were found between the sex of infants in the linear regression analysis by MaAsLin2. This again indicates that probiotic supplementation may be responsible for the increase in *Lactobacillaceae* but not sex itself.

Calprotectin levels decreased with increasing postnatal age (Supplementary Figure S9). These observations were similar to previous studies of preterm infants. A positive correlation between *Clostridium* and *Staphylococcus* bacteria and calprotectin was noted in a previous study, however no such a correlation observed in this study [29]. Further studies are required to confirm these findings.

Probiotic supplementation increases the synthesis of the bile acid metabolites, cholate and taurine, leading to the increased intestinal absorption of lipids in preterm infants [30]. The functional prediction analysis in the current study is consistent with previous findings that the genes involved in primary and secondary bile acids are elevated in preterm infants supplemented with probiotics.

The limitations of this study include that the effects of antibiotic exposure cannot be assessed. All the infants received antibiotics at any point of time in the NICU without exception, and 95% of them received broad-spectrum antibiotics, including meropenem and colistin. In addition, only a few babies received donor milk. The duration of EBM and donor milk intake was varies in babies and ranges from a few days up to weeks. This makes it difficult to interpret the effect of time and volume of EBM and donor milk on microbiota changes in infants.

In the current study, we showed that probiotics were the most significant factor responsible for intestinal microbial changes in preterm neonates. Similar observations found in the intervention studies of probiotics supplementation compared to the placebo arm showed that probiotics drive a major alteration of microbial composition compared to other factors, including postnatal age, sex, gestational age, birth weight, breast milk, antibiotics, and mode of delivery [30,31]. In fact, in the present study, additional covariates, including calprotectin and time to full feed, did not show any association with bacterial composition.

In conclusion, probiotic supplementation makes a significant contribution to altering the gut microbial composition in premature infants admitted to the NICU. Further studies are required to reveal the role of probiotics in the alteration of microbial and host metabolic genes in preterm infants.

## Figures and Tables

**Table 1:** Demographic details of subjects.

Variables	n = 16
Gestational age (week), mean ± SD	28.3 ± 1.5
Sex (male/female)	7/9
Birth weight (g), mean ± SD	1090 ± 219
No. (%) of extreme preterm subjects (≤28 weeks)	7 (43.8%)
No. (%) of Vaginal/Cesarean section	9 (56.25%)/(743.8%)
No. (%) of mothers with Preterm Premature rupture of membranes	9 (56.25%)
No. (%) of mothers with preeclampsia	2 (12.5%)
No. (%) of mothers who received antenatal corticosteroids	15 (93.8%)
APGAR score at 1 min, median (range)	6 (4–8)
APGAR score at 5 min, median (range)	8 (7–10)
No. (%) of neonates receiving antibiotics	15 (93.8%)
Media (range) days taken for full feed (120 mL/kg/d)	12 (5–24)

**Table 2:** PERMANOVA multivariate analysis performed based on Bray–Curtis dissimilarity distance. Week—samples collected weekly once until four weeks of life.

	Df	Sums of Sqs	Mean Sqs	F.Model	R2	Pr(>F)
Week	3	0.543	0.543	2.563	0.030	0.017
Probiotics	1	0.758	0.758	3.579	0.042	0.004
Preterm	1	0.619	0.619	2.919	0.035	0.015
Birth weight	1	0.503	0.503	2.372	0.028	0.027
Mode of delivery	1	0.486	0.486	2.296	0.027	0.027
Sex	1	0.568	0.568	2.680	0.032	0.023
Week:Probiotics	3	0.426	0.426	2.009	0.024	0.054
Week:Preterm	3	0.194	0.194	0.916	0.011	0.501
Probiotics:Preterm	1	0.250	0.250	1.181	0.014	0.311
Week:Birth weight	3	0.378	0.378	1.786	0.021	0.082
Probiotics:Birth weight	1	0.143	0.143	0.673	0.008	0.706
Preterm: Birth weight	1	0.660	0.660	3.115	0.037	0.006
Week:Mode of delivery	3	0.182	0.182	0.858	0.010	0.538
Probiotics: Mode of delivery	1	0.119	0.119	0.561	0.007	0.821
Preterm: Mode of delivery	1	0.752	0.752	3.548	0.042	0.007
Week:sex	3	0.210	0.210	0.992	0.012	0.421
Probiotics:sex	1	0.319	0.319	1.504	0.018	0.158
Preterm:sex	1	0.618	0.618	2.918	0.035	0.01
Birth weight:sex	1	1.198	1.198	5.654	0.067	0.001
Mode of delivery:sex	1	0.166	0.166	0.784	0.009	0.582
Week:Probiotics:Preterm	3	0.205	0.205	0.967	0.011	0.407
Week:Preterm: Birth weight	3	0.246	0.246	1.160	0.014	0.314
Probiotics:Preterm: Birth weight	3	0.212	0.212	0.999	0.012	0.45
Week:Probiotics:Mode of delivery	2	0.175	0.175	0.826	0.010	0.587
Week:Preterm:Mode of delivery	3	0.236	0.236	1.113	0.013	0.317
Probiotics:Preterm:Mode of delivery	1	0.176	0.176	0.833	0.010	0.593
Week:Probiotics:sex	1	0.129	0.129	0.607	0.007	0.758
Week:Preterm:sex	3	0.160	0.160	0.754	0.009	0.618
Probiotics:Preterm:sex	1	0.227	0.227	1.073	0.013	0.346
Week: Birth weight:sex	1	0.138	0.138	0.651	0.008	0.721
Week: Mode of delivery: sex	1	0.399	0.399	1.884	0.022	0.085
Probiotics: Mode of delivery: sex	1	0.616	0.616	2.906	0.034	0.009
Week:Probiotics:Preterm: Mode of delivery	1	0.225	0.225	1.060	0.013	0.362
Week:Probiotics:Preterm:sex	1	0.135	0.135	0.636	0.008	0.749
Residuals	26	5.510	0.212	NA	0.308	NA
Total	60	17.880	NA	NA	1.000	NA

**Table 3:** Microbial taxa significantly associated with probiotic supplementation identified by MaAsLin2 analysis.

Feature	Metadata	Regression Coefficient	Standard Error	*p* Value	BH-Adjusted *p* Value
Enterobacteriaceae	Probiotics	3.145	0.804	0.000	0.062
Enterobacteriaceae.1	Probiotics	2.849	0.773	0.001	0.063
Klebsiella.unclassified	Probiotics	3.081	0.967	0.002	0.084
Klebsiella.unclassified.1	Probiotics	2.963	0.915	0.002	0.084
Staphylococcus.unclassified	Probiotics	2.475	0.779	0.002	0.084
Staphylococcus.unclassified.1	Probiotics	2.622	0.824	0.002	0.084
Enterobacteriaceae.9	Probiotics	2.292	0.668	0.001	0.084
Bifidobacterium.longum	Probiotics	2.360	0.834	0.006	0.127
Bifidobacterium.unclassified	Probiotics	2.326	0.831	0.007	0.127
Bacteroides.unclassified	Probiotics	1.060	0.364	0.005	0.127
Bacteroides.vulgatus	Probiotics	1.067	0.365	0.005	0.127
Klebsiella.unclassified.3	Probiotics	2.615	0.946	0.008	0.127
Klebsiella.unclassified.4	Probiotics	2.216	0.801	0.008	0.127
Escherichia.Shigella.unclassified.2	Probiotics	1.914	0.685	0.007	0.127
Escherichia.Shigella.unclassified.5	Probiotics	2.521	0.914	0.008	0.127
Klebsiella.unclassified.2	Probiotics	2.268	0.852	0.010	0.152
Acinetobacter.unclassified.2	Probiotics	−1.448	0.548	0.011	0.152
Klebsiella.unclassified.5	Probiotics	2.970	1.152	0.013	0.173
Enterobacterales	Probiotics	1.644	0.665	0.016	0.202
Enterobacteriaceae.14	Probiotics	1.657	0.675	0.017	0.202
Lacticaseibacillus.unclassified	Probiotics	1.960	0.801	0.018	0.202
Enterobacteriaceae.15	Probiotics	2.180	0.906	0.020	0.217
Acinetobacter.unclassified.3	Probiotics	−1.363	0.580	0.022	0.234
Bifidobacterium.longum.1	Probiotics	1.461	0.634	0.025	0.245
Bifidobacterium.unclassified.1	Probiotics	1.524	0.657	0.025	0.245

**Table 4:** Microbial taxa significantly altered in samples collected from the first to fourth weeks identified by the MaAsLin2 analysis.

Feature	Metadata	Regression Coefficient	Standard Error	*p*-Value	BH-Adjusted *p* Value
Klebsiella.unclassified.2	Week	1.22908	0.321885	0.000417	0.020781
Klebsiella.unclassified.3	Week	1.41849	0.355709	0.000248	0.020781
Enterobacteriaceae	Week	1.186119	0.331487	0.000839	0.020781
Clostridium.sensu.stricto.1.unclassified.1	Week	0.960771	0.269656	0.000846	0.020781
Enterobacteriaceae.15	Week	1.22519	0.342688	0.000859	0.020781
Klebsiella.unclassified	Week	1.346191	0.388773	0.00119	0.022955
Klebsiella.unclassified.1	Week	1.25157	0.369885	0.001499	0.022955
Enterobacteriaceae.13	Week	1.079414	0.320783	0.001518	0.022955
Enterobacteriaceae.1	Week	1.050946	0.319754	0.001958	0.026326
Staphylococcus.unclassified.7	Week	0.416346	0.128861	0.002339	0.028307
Enterobacteriaceae.16	Week	0.644443	0.222827	0.005917	0.047907
Bifidobacterium.unclassified.1	Week	0.712908	0.254997	0.007618	0.053069
Escherichia.Shigella.unclassified.5	Week	1.049388	0.37369	0.007291	0.053069
Enterobacteriaceae.9	Week	0.776773	0.280789	0.008193	0.053069
Enterococcus.unclassified.18	Week	−0.8197	0.297401	0.008333	0.053069
Bifidobacterium.longum.1	Week	0.672177	0.246387	0.009087	0.054978
Escherichia.Shigella.unclassified.2	Week	0.730768	0.281493	0.012638	0.069508
Bifidobacterium.breve	Week	0.679655	0.26794	0.014749	0.071723
Bifidobacterium.unclassified.2	Week	0.804917	0.317121	0.014693	0.071723
Streptococcus.unclassified.6	Week	0.402735	0.160303	0.015801	0.071723
Klebsiella.unclassified.4	Week	0.769098	0.327439	0.02333	0.095271
Veillonella.unclassified.4	Week	0.434013	0.186852	0.024408	0.095271

## Data Availability

Sequencing data available in the Sequence Read Archive (SRA) portal (Submission ID: SUB15044229 and BioProject ID: PRJNA1216735),https://www.ncbi.nlm.nih.gov/bioproject/?term=PRJNA1216735, accessed on 30 January 2025.

## Supplementary Materials

The following supporting information can be downloaded at: https://www.mdpi.com/article/10.3390/microorganisms13030577/s1, Figure S1. Alpha diversity metrics, observed ASVs (1a) and Shannon diversity (1b) on C-section and vaginal delivery; Figure S2. Observed ASVs (a) and Shannon diversity (b) on extreme preterm (Gestation age <28 weeks) and very preterm infants (Gestation age 28 to 32 weeks); Figure S3. Observed ASVs (a) and Shannon diversity (b) on birth weight of <1000 g and >1000 g; Figure S4. LEfSe analysis for differential microbial abundance between very preterm infants and extreme preterm infants. Bacterial abundance shown only to significant corresponding Taxa level; Figure S5. LEfSe analysis for differential microbial abundance between preterm infants weight above 1000 g compared to low birth weight; Figure S6. LEfSe analysis for differential microbial abundance between preterm infants born by C-section and vaginal delivery; Figure S7. LEfSe analysis identified differential microbial abundance between samples collected in the first four weeks of life; Figure S8. Microbial taxa significantly associated with probiotic supplementation identified by MaAsLin2 analysis; Figure S9. Fecal Calprotectin (ug/g) levels in preterm infants from the first four weeks of life; Table S1. PERMANOVA multivariate analysis performed based on Jaccard dissimilarity distance; Table S2. PERMANOVA multivariate analysis performed based on weighted Unifrac distance; Table S3. LEfSe analysis identified significant metabolic pathways in samples of probiotic supplementation compared to non-supplemented groups.
