# Supplementary material for: Gut Microbiota Alteration in Healthy Preterm Infants: An Observational Study from Tertiary Care Center in India
Source: Microorganisms. 2025 Mar 3;13(3):577. doi: 10.3390/microorganisms13030577 (PMC11944540; doi:10.3390/microorganisms13030577)
Supplement: Supplementary file 1 [file microorganisms-13-00577-s001.zip › microorganisms-3452296-supplementary.pdf]

Supplementary Material

Table S1. PERMANOVA multivariate analysis performed based on Jaccard dissimilarity distance

	Df	SumsOfSqs	MeanSqs	F.Model	R2	Pr(>F)
Week	3	0.60499	0.60499	2.266383	0.02805	0.012
Probiotics	1	0.719906	0.719906	2.696876	0.033378	0.003
Preterm	1	0.755943	0.755943	2.831879	0.035048	0.003
Birth_Weight	1	0.575504	0.575504	2.155924	0.026683	0.013
Mode_of_Delivery	1	0.511123	0.511123	1.914744	0.023698	0.027
Gender	1	0.59386	0.59386	2.224692	0.027534	0.013
Week:Probiotics	3	0.507013	0.507013	1.899349	0.023507	0.029
Week:Preterm	1	0.308943	0.308943	1.157349	0.014324	0.274
Probiotics:Preterm	1	0.298158	0.298158	1.116944	0.013824	0.308
Week:Birth_Weight	3	0.442461	0.442461	1.657527	0.020514	0.059
Probiotics:Birth_Weight	1	0.237056	0.237056	0.888046	0.010991	0.562
Preterm:Birth_Weight	1	0.723107	0.723107	2.708867	0.033526	0.002
Week:Mode_of_Delivery	3	0.264386	0.264386	0.990429	0.012258	0.454
Probiotics:Mode_of_Delivery	1	0.218995	0.218995	0.820388	0.010153	0.66
Preterm:Mode_of_Delivery	1	0.751169	0.751169	2.813993	0.034827	0.001
Week:Gender	3	0.30193	0.30193	1.131077	0.013999	0.28
Probiotics:Gender	1	0.381766	0.381766	1.430155	0.0177	0.11
Preterm:Gender	1	0.625953	0.625953	2.344914	0.029022	0.008
Birth_Weight:Gender	1	1.138115	1.138115	4.263552	0.052767	0.001
Mode_of_Delivery:Gender	1	0.477129	0.477129	1.787399	0.022122	0.042
Week:Probiotics:Preterm	3	0.26365	0.26365	0.987674	0.012224	0.427
Week:Preterm:Birth_Weight	3	0.313617	0.313617	1.174858	0.01454	0.25
Probiotics:Preterm:Birth_Weight	1	0.265966	0.265966	0.996351	0.012331	0.416
Week:Probiotics:Mode_of_Delivery	3	0.275874	0.275874	1.033467	0.012791	0.405
Week:Preterm:Mode_of_Delivery	3	0.319808	0.319808	1.198049	0.014828	0.238
Probiotics:Preterm:Mode_of_Delivery	1	0.256805	0.256805	0.96203	0.011906	0.453
Week:Probiotics:Gender	3	0.21046	0.21046	0.788417	0.009758	0.695
Week:Preterm:Gender	3	0.266392	0.266392	0.997946	0.012351	0.406
Probiotics:Preterm:Gender	1	0.360425	0.360425	1.350205	0.016711	0.139
Week:Birth_Weight:Gender	3	0.203949	0.203949	0.764025	0.009456	0.716
Week:Mode_of_Delivery:Gender	3	0.411251	0.411251	1.54061	0.019067	0.091
Probiotics:Mode_of_Delivery:Gender	1	0.563279	0.563279	2.110128	0.026116	0.011
Week:Probiotics:Preterm:Mode_of_Delivery	3	0.293059	0.293059	1.097843	0.013587	0.316
Week:Probiotics:Preterm:Gender	1	0.186064	0.186064	0.697026	0.008627	0.808
Residuals	26	6.940455	0.266941	NA	0.321786	NA
Total	60	21.56856	NA	NA	1	NA

Table S2. PERMANOVA multivariate analysis performed based on weighted Unifrac distance

	**Df**	**SumOfSqs**	**R2**	**F**	**Pr(>F)**
Week	3	0.04034	0.03599	2.2029	**0.056**
Probiotics	1	0.06233	0.05561	3.4742	**0.01**
Preterm	1	0.05104	0.04554	2.8149	**0.026**
Birth_Weight	1	0.05376	0.04797	2.9725	**0.019**
Mode_of_Delivery	1	0.04138	0.03692	2.2616	**0.077**
Gender	1	0.01928	0.0172	1.0325	0.383

Table S3. LEfSe analysis identified significant metabolic pathways in samples of probiotic supplementation compared to non-supplemented groups.

feature	p_values	adj_method	p_adjust	pathway_name	pathway_class	pathway_map
ko00760	0.001148	BH	0.048825	Nicotinate and nicotinamide metabolism	Metabolism; Metabolism of cofactors and vitamins	Nicotinate and nicotinamide metabolism
ko00720	0.000849	BH	0.048825	Other carbon fixation pathways	Metabolism; Energy metabolism	Other carbon fixation pathways
ko00790	0.001298	BH	0.048825	Folate biosynthesis	Metabolism; Metabolism of cofactors and vitamins	Folate biosynthesis
ko00660	0.001457	BH	0.048825	C5-Branched dibasic acid metabolism	Metabolism; Carbohydrate metabolism	C5-Branched dibasic acid metabolism
ko05100	0.000173	BH	0.017377	Bacterial invasion of epithelial cells	Human Diseases; Infectious disease: bacterial	Bacterial invasion of epithelial cells
ko04144	1.31E-09	BH	2.64E-07	Endocytosis	Cellular Processes; Transport and catabolism	Endocytosis

1a

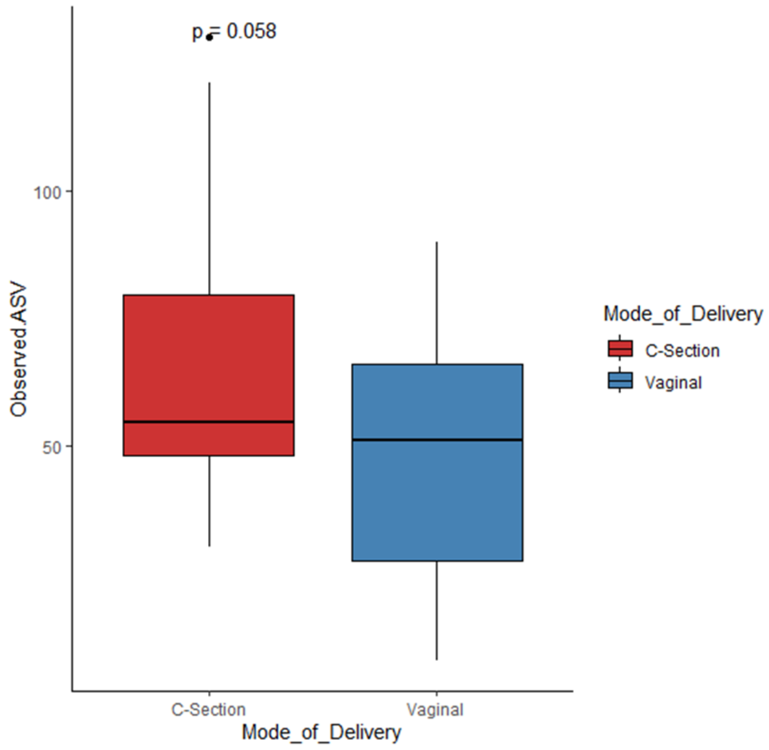

1b

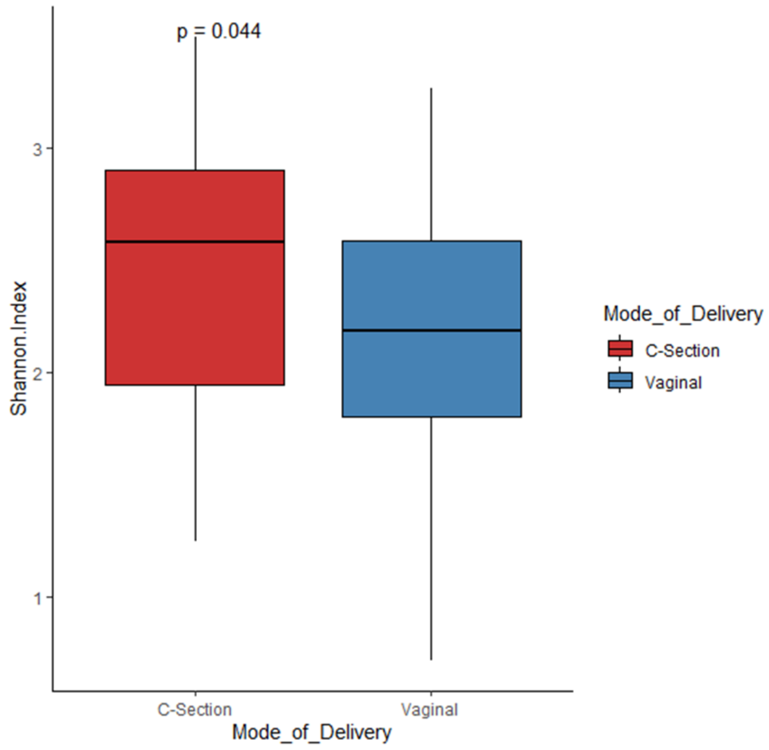

Figure S1. Alpha diversity metrics, observed ASVs (1a) and Shannon diversity (1b) on C-section and vaginal delivery.

2a

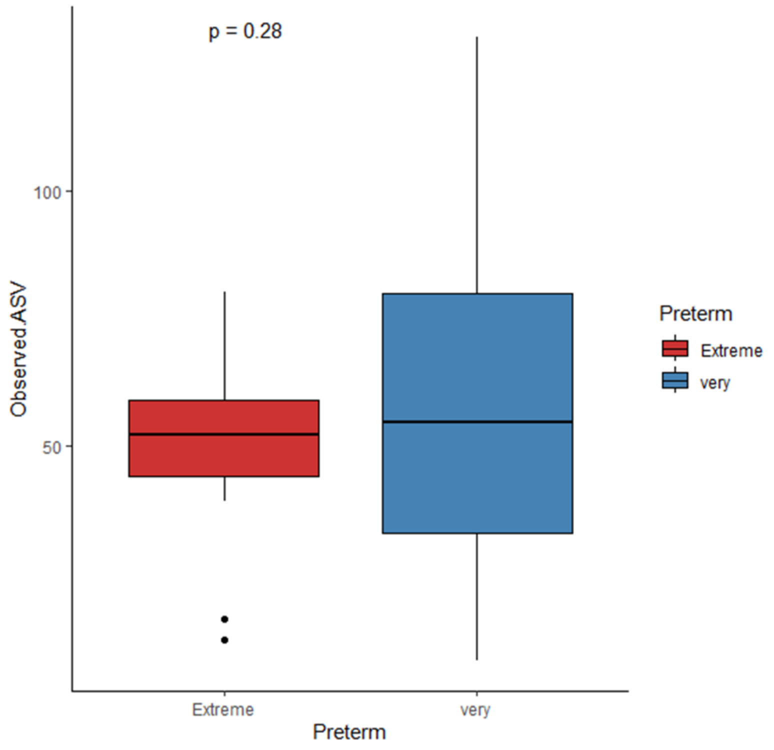

2b

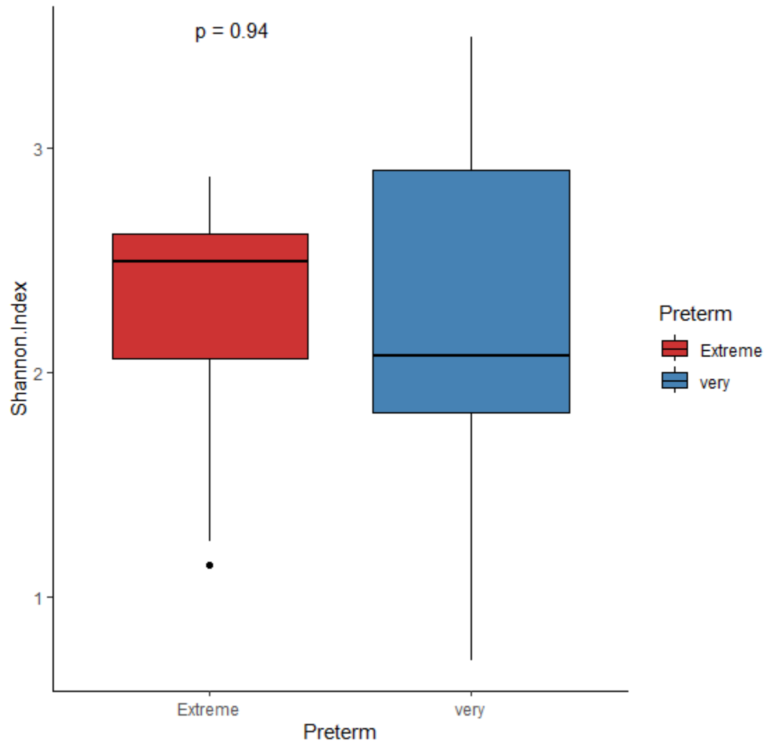

Figure S2. Observed ASVs (a) and Shannon diversity (b) on extreme preterm (Gestation age <28 weeks) and very preterm infants (Gestation age 28 to 32 weeks).

3a

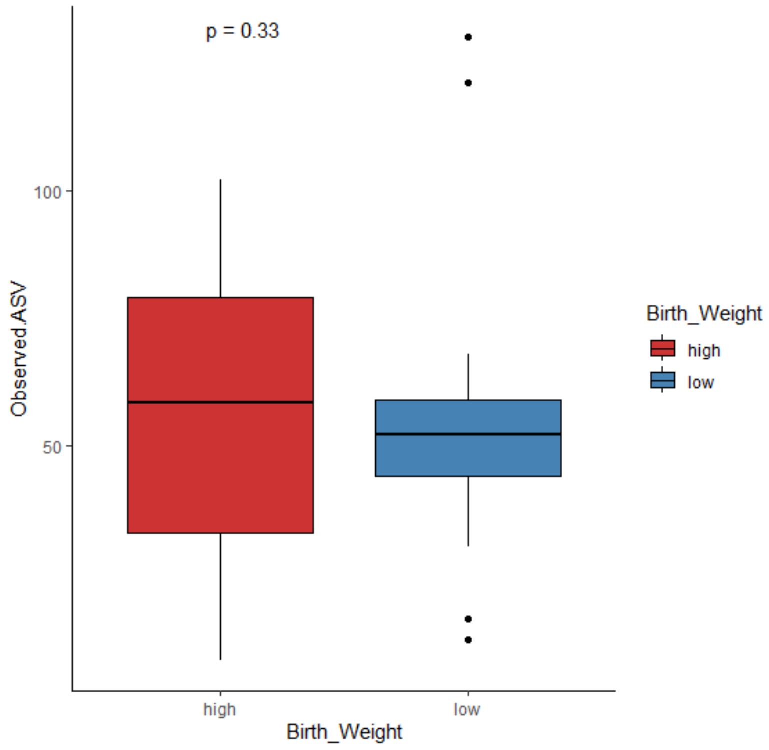

3b

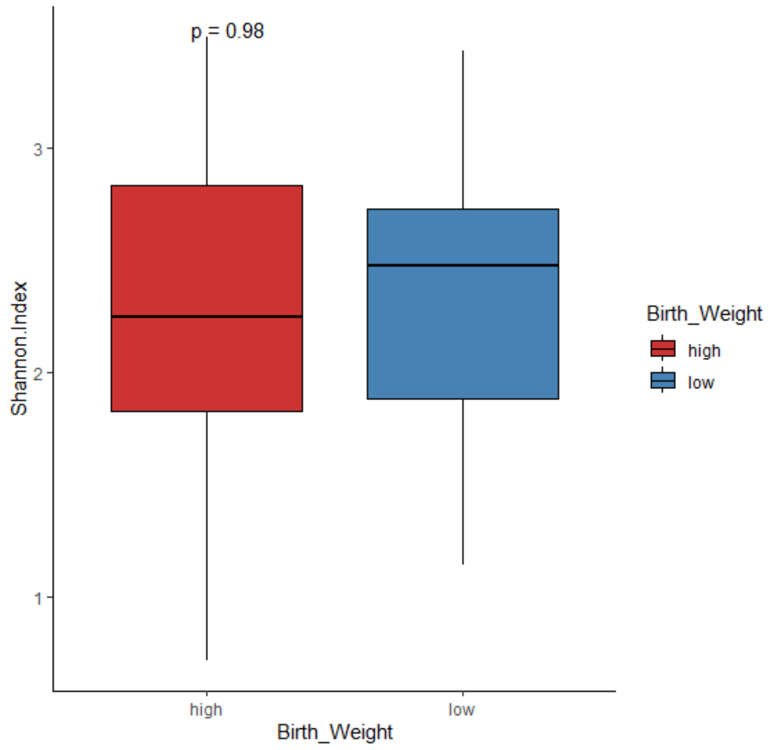

Figure S3. Observed ASVs (a) and Shannon diversity (b) on birth weight of <1000gram and >1000gram.

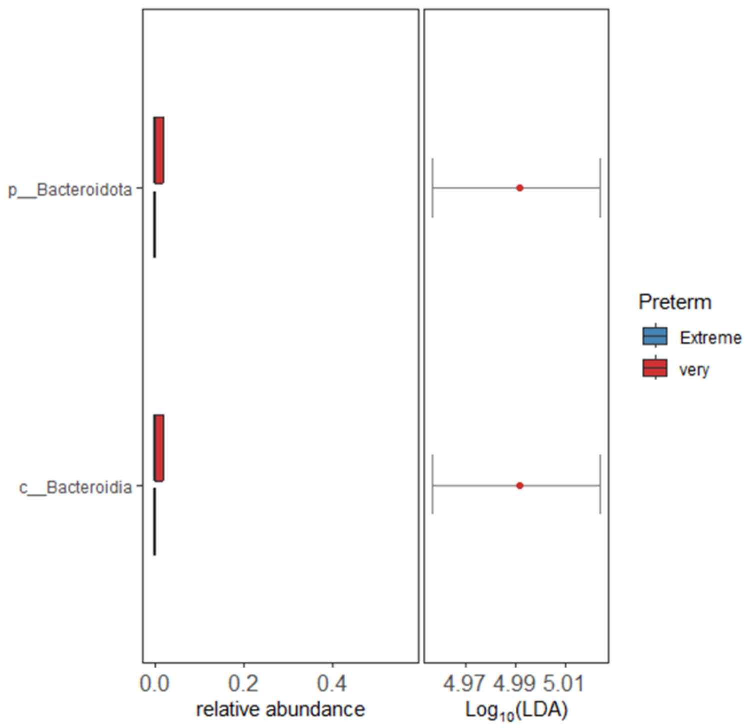

Figure S4. **LEfSe analysis for differential microbial abundance between very preterm infants and extreme preterm infants.** Bacterial abundance shown only to significant corresponding Taxa level.

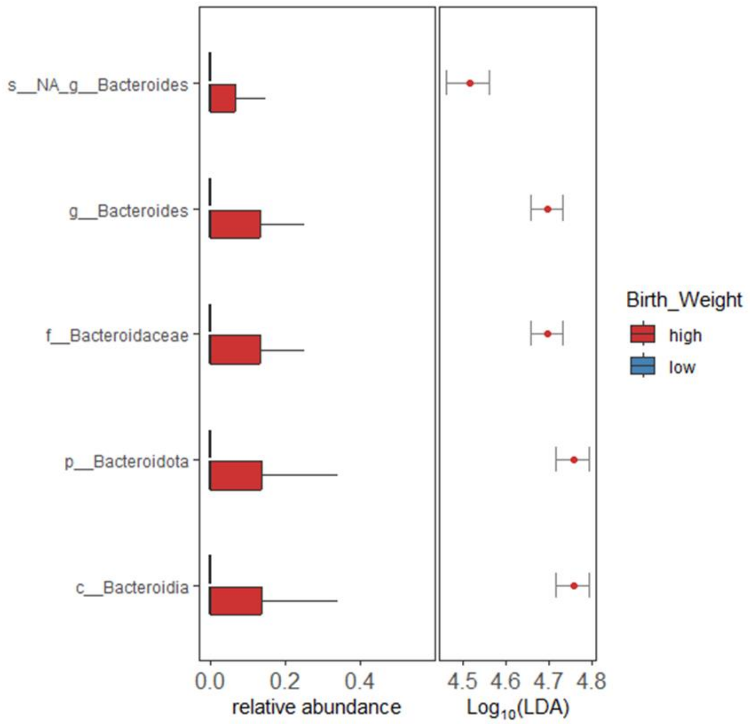

Figure S5. **LEfSe analysis for differential microbial abundance between preterm infants weight above 1000 gm compared to low birth weight**

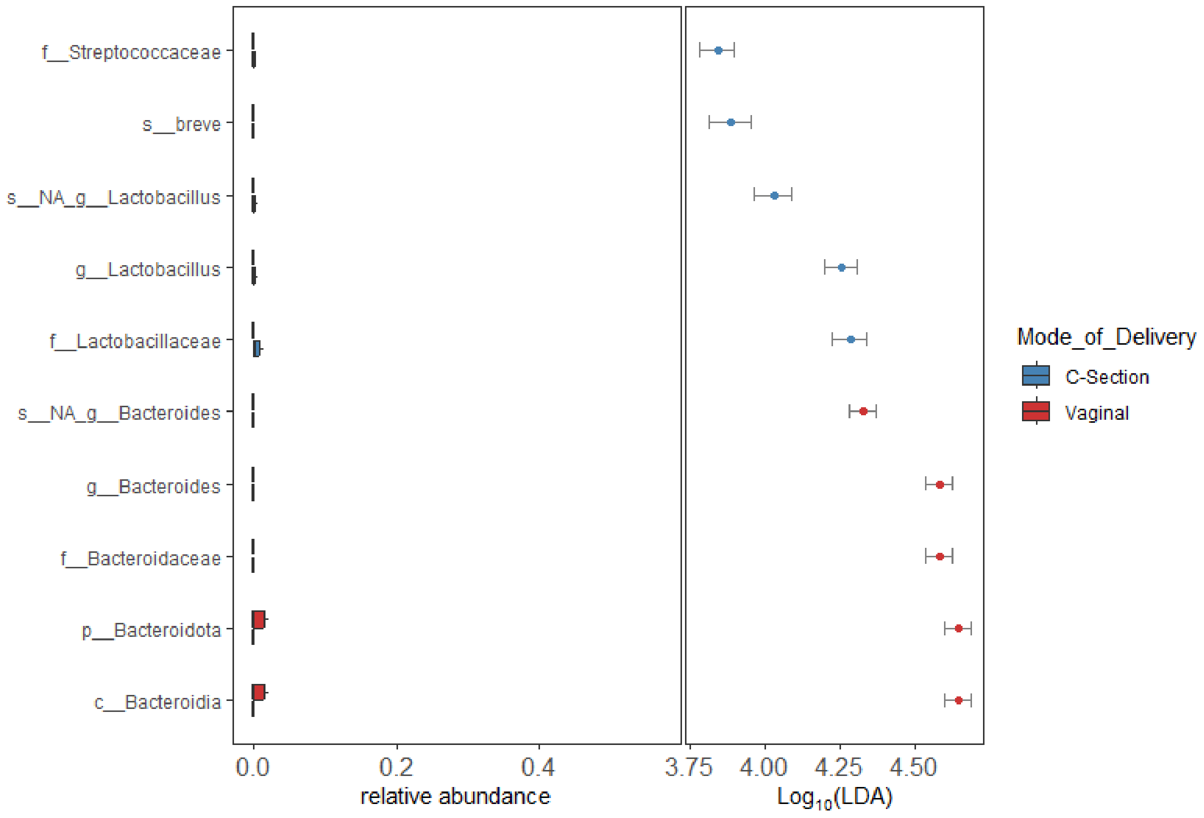

Figure S6. LEfSe analysis for differential microbial abundance between preterm infants born by C-section and vaginal delivery.

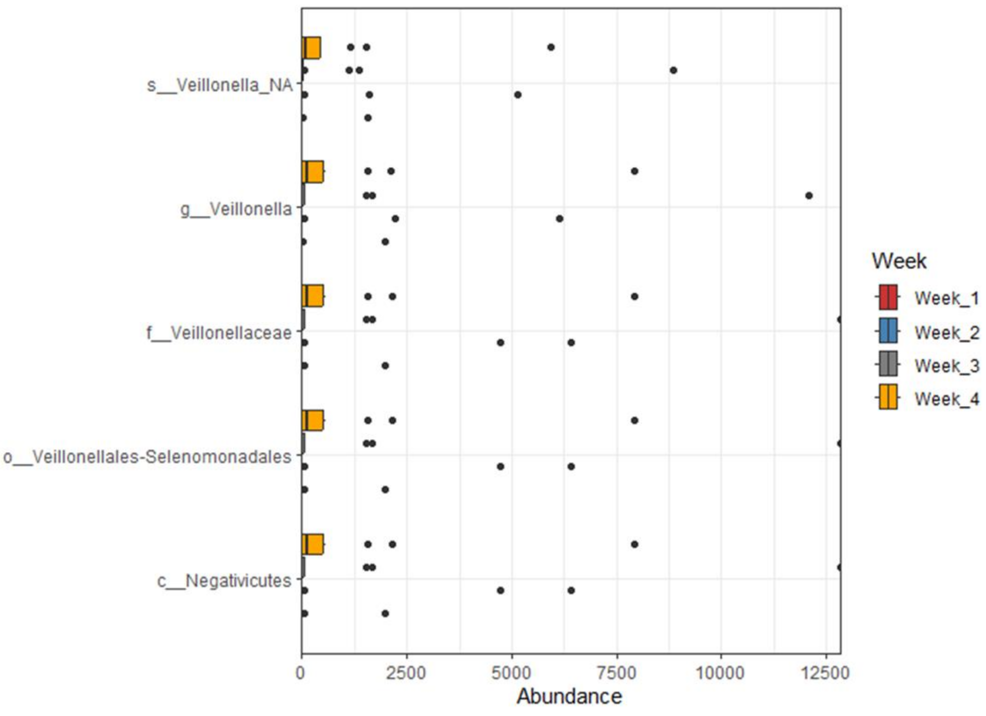

Figure S7. LEfSe analysis identified differential microbial abundance between samples collected in the first four weeks of life.

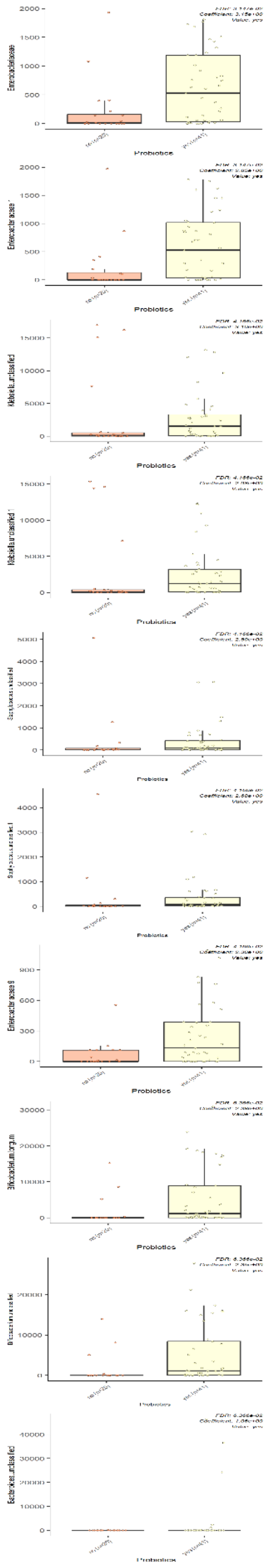

Figure S8. Microbial taxa significantly associated with probiotic supplementation identified by MaAsLin2 analysis.

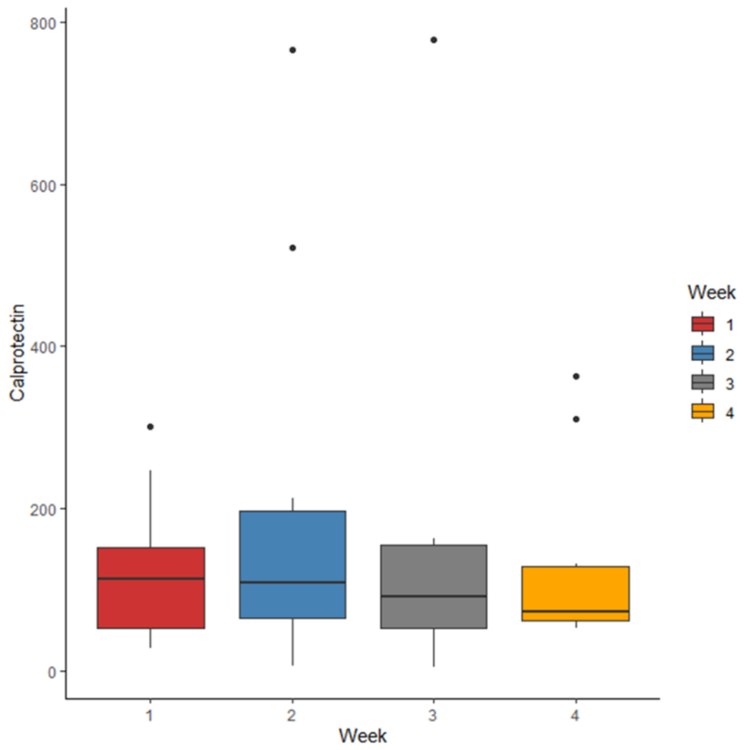

Figure S9. Fecal Calprotectin (ug/g) levels in preterm infants from the first four weeks of life.
